# Investigating the Sensory, Nutritional, and Volatile Properties of Two Local Sweet Cherry (*Prunus avium* L.) Cultivars From Central Italy

**DOI:** 10.1155/ijfo/7212819

**Published:** 2026-07-03

**Authors:** Maria Teresa Frangipane, Lara Costantini, Stefania Garzoli, Nicolò Merendino, Saverio Senni, Riccardo Massantini

**Affiliations:** ^1^ Dipartimento per la Innovazione nei sistemi Biologici Agroalimentari e Forestali, Università degli Studi della Tuscia, Viterbo, Lazio, Italy, unitus.it; ^2^ Università degli Studi della Tuscia, Dipartimento di Scienze Ecologiche e Biologiche, Viterbo, Lazio, Italy, unitus.it; ^3^ Dipartimento di Chimica e Tecnologia del Farmaco, Università degli Studi di Roma La Sapienza, Rome, Lazio, Italy, uniroma1.it; ^4^ Dipartimento di Scienze Agrarie e Forestali, Università degli Studi della Tuscia, Viterbo, Lazio, Italy, unitus.it; ^5^ Universita degli Studi della Tuscia, Viterbo, Lazio, Italy

**Keywords:** antioxidants, cherries, quality, sensorial traits, VOCs

## Abstract

The antioxidant activity, sensory profile, and volatile composition of two native cherry cultivars grown in the Celleno area of central Italy were studied for the first time. This was done to assess the potential for valorizing this product, both for its excellent flavor and its potential as a source of bioactive compounds. The two cultivars stood out for their complex aromas, each with distinct descriptive notes. The “Ravenna a gambo lungo” cherry exhibited high levels of cherry (9.08 ± 0.53), apple (1.95 ± 0.50), and floral (3.43 ± 1.01) aromas, as well as high aromatic intensity (9.09 ± 0.34). On the other hand, the “Graffione” cherries showed higher sweetness (8.48 ± 0.55) and a plum aroma (9.05 ± 0.53). Despite the fact that both cultivars under scrutiny received elevated scores in the overall judgment, the “Ravenna a gambo lungo” cultivar achieved a higher score (9.07 ± 0.52) than the “Graffione” cultivar (8.05 ± 0.50). No significant differences were found between the two samples in terms of antioxidant activity. Both samples exhibited high antioxidant capacity, equivalent to TPC (3.27 and 3.26 mg GAE/g for “Ravenna a gambo lungo” and “Graffione,” respectively), ABTS^•+^ (1.20 and 1.25 mmol TE/g for “Ravenna a gambo lungo” and “Graffione,” respectively) and FRAP (2.26 and 2.14 mmol Fe^2+^/g for “Ravenna a gambo lungo” and “Graffione,” respectively), rendering them nutritionally valuable. Significant differences were also detected between the two cherry samples analyzed in terms of volatile compounds. The main volatiles in “Ravenna a gambo lungo” cherries were trans‐2‐hexenol (38.9%), decanal (22.1%), nonanal (8.9%), benzaldehyde (7.5%), and cis‐geranyl acetate (6.3%). In “Graffione,” the most abundant volatiles were linalool (24.7%) and hexanal (16.4%).

## 1. Introduction

**Figure 1:**
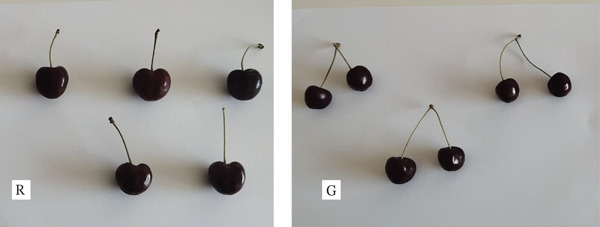
Sweet cherries of the two analyzed cultivars: G, “Graffione”; R, “Ravenna a gambo lungo.”

The sweet cherry (*Prunus avium* L.) is one of the most commonly grown fruits in temperate climates. According to data from the Food and Agriculture Organization (FAO) (2025), global sweet cherry production is estimated at 2,963,780 t. Turkey is the world′s leading producer (736,791 t), followed by Chile (465,348 t) and the United States (321,420 t). Italy is the sixth‐largest producer in the world, with a cherry production of 87,710 t [1]. Sweet cherries are a highly valuable crop, both economically and nutritionally, thanks to the health benefits and commercial potential of their fruits. Characterized by their bright red color and pleasant flavor, they have earned the nickname “the jewels of the fruit” [2]. Their high nutritional value, much appreciated by consumers, is due to the presence of sugars, proteins, vitamins, calcium, iron, and a rich polyphenolic content, which confer numerous beneficial effects on human health [3–5]. In this regard, recent studies have demonstrated that consuming diets enriched with cherries reduces proinflammatory molecules, as well as triglyceride and cholesterol levels [6, 7]. In particular, it is the bioactive compounds, such as flavonoids and anthocyanins, that determine the antioxidant capacity of sweet cherries. These compounds play a therapeutic role in human health and affect the quality, taste, and flavor of the fruit [8]. Kelebek and Selli [9] reported that the total anthocyanin content ranged from 47.10 mg/100 g of fresh weight (FW) in the Van cultivar (cv.) to 142.64 mg/100 g of FW in the Noir de Guben cv. Furthermore, Kelebek and Selli [9] discovered a significant correlation between antioxidant capacity and phenolic content, suggesting that phenolic compounds are the primary contributors to the antioxidant properties of sweet cherries. On the other hand, phenolic compounds are highly dependent on genetic variability. To obtain high‐quality products, it is advisable to select the most suitable cultivars for the territory′s environmental and geomorphological characteristics. “Local varieties” are those that have been selected and cultivated over time for their ability to adapt to specific geomorphological characteristics found in certain territories [10]. These cultivars are a fundamental asset for agrobiodiversity and the sustainability of production systems [11]. The Mediterranean region is a particularly fruitful area for local varieties of sweet cherry, especially in Italy, including the Lazio region, where the fruits examined in this study were collected. Particularly, Celleno, in the province of Viterbo, has a historic vocation to produce cherries. The Municipal Statute of 1457 already mentions the presence of “cherries” in local agriculture when it established penalties for those who stole them from other people′s fields [12]. In the agricultural economy of the past century, sweet cherry cultivation expanded throughout the area, assuming particular importance for the sale of the fruit. Peasant families earned their first income after the winter, when there were no produce sales. The modernization of the sector then led to a partial abandonment of the plantations until the early 2000s. The establishment of the Celleno Cherry Consortium revived interest in the crop, and new plantations were established, managed with modern agronomic techniques. In this scenario, our objective was to deliberate innovative insights with a focus on the sensory and nutritional characteristics of two Italian local sweet cherry cultivars, “Graffione” and “Ravenna a gambo lungo” (Figure 1).

They are local cultivars from the provinces of Rieti, Rome, and Viterbo (Lazio region, Italy), registered with Officially Recognized Description (ORD) in the National Register of Varieties of fruit plants allowed for marketing [13]. Favoring local cherry varieties not only protects biodiversity but also allows for greater resistance to any adverse weather events. Studying local cherry varieties is fundamental to protecting biodiversity and managing agriculture sustainably [14]. Preserving them is also important because local varieties represent a territory′s heritage of culture and tradition.

## 2. Materials and Methods

### 2.1. Sample Preparation

Samples of the local sweet cherry cultivars “Graffione” and “Ravenna a gambo lungo,” weighing 5 kg each cultivar, were collected in June 2025 in Celleno, a village in the province of Viterbo situated about 364 m above sea level (42°54 ^′^69 ^″^ N, 12°13 ^′^32 ^″^ E). The sweet cherries came from the “Consortium for the Protection and Enhancement of Celleno Cherries,” whose objectives include improving local sweet cherry cultivars, with the aim of establishing them as typical and excellent products of the Lazio region of Italy. The quality of the samples was initially assessed by visual inspection, with any defective samples being excluded. Three replicates were measured for each cultivar, with 30 fruits used to measure each parameter in each replicate.

### 2.2. Morphological Properties

The morphological assessment of each fruit began with the determination of its basic linear dimensions—namely length (L), width (W), and thickness (T)—using a digital caliper with a measurement resolution of 0.01 mm. From these primary data, secondary geometric characteristics such as fruit volume, geometric mean diameter (Dg), surface area (S), and sphericity (*Ø*) were derived using the computational approach described by Mohsenin [15]. Individual fruit mass was obtained with an electronic balance having a precision of ± 0.001 g after proper calibration. In addition, firmness was quantified at the equatorial zone of each fruit by measuring the peak compression force with an AGROSTA100 portable digital firmness tester.

### 2.3. Sensory Analysis

The sensory study involved a group of eight trained assessors, coordinated by a designated panel leader [16]. Prior to the evaluation phase, the panel engaged in three structured training sessions aimed at aligning individual perceptions and establishing a coherent descriptive framework. Throughout these sessions, the assessors jointly discussed, selected, and refined the terminology to ensure consistent interpretation of sensory attributes [2, 17, 18]. All analyses were performed in a purpose‐built sensory facility designed to meet ISO 8589 requirements [19]. The intensity of each attribute was quantified using a 0–10 rating system, where the lower endpoint indicated no perceptible sensation and the upper endpoint corresponded to the highest detectable intensity.

Five samples of each cherry cultivar were presented to the panelists in a random order and were identified by a unique three‐digit code. Prior to evaluation, the samples were left to reach room temperature (approximately 18°C) for approximately 2 h. To maintain consistency in presentation, all samples were served in white plastic dishes. Mineral water was made available to panelists for palate cleansing between samples. All judges were nonsmokers and refrained from wearing perfume or consuming food or beverages that could affect their sensory perception for at least 1 h prior to the evaluation. In addition to specific sensory attributes, panel members expressed an overall preference score for each sample using a subjective hedonic scale ranging from 0 to 10. All activities involving human participants were carried out in compliance with relevant UNI EN ISO [16] and ISO [19] standards governing sensory analysis. The panel was officially accredited by the Italian Ministry of Agriculture, Food Sovereignty and Forestry, and all participants provided written informed consent prior to involvement.

### 2.4. Extracts′ Preparation for Polyphenol Compounds and Antioxidant Activity Determination

Edible samples were ground and extracted overnight in the dark with 80% methanol (1:25, *w*/*v*) following Costantini et al. [20]. Extracts were centrifuged (10,000 rpm, 10 min, 4°C; ALC PK121R centrifuge; Bodanchimica s.r.l., Cagliari, Italy), and the supernatant was used for analysis.

### 2.5. Total Phenolic Content (TPC)

TPC was determined by the Folin–Ciocalteu method [20], adapted to microplates. Briefly, 10‐*μ*L extract, 30‐*μ*L water, 10‐*μ*L reagent, and 200‐*μ*L 30% Na_2_CO_3_ were mixed. Absorbance was read at 725 nm after 30‐min incubation (Infinite F200, Tecan, Switzerland). Results were expressed as milligrams of gallic acid equivalents per gram..

### 2.6. Total Antioxidant Capacity (TAC) Determination

TAC was evaluated by FRAP and ABTS assays.

FRAP was performed as described by Benzie and Strain [21], adapted to 96‐well plates. The reaction mixture (FRAP reagent and sample) was incubated (37°C, 30 min) and read at 595 nm (Infinite 2000, Tecan, Salzburg, Austria). Results were expressed as millimoles of ferrous iron per gram.

ABTS^•+^ activity was measured using a TEAC kit (Cell Biolabs Inc.) according to the manufacturer′s protocol. Absorbance was read at 405 nm, and results were expressed as millimoles of Trolox equivalents per gram.

### 2.7. HS‐SPME/GC‐MS Analysis of Volatile Fraction

The volatile profile of cherry samples was described using headspace solid‐phase microextraction (HS‐SPME) coupled with gas chromatography–mass spectrometry (GC‐MS). In brief, approximately 0.5 g of the sample was transferred to a 7‐mL vial that was sealed with a PTFE/silicone septum, to which a sodium chloride solution was added to enhance the release of the volatiles. Extraction was performed using a 1‐cm Supelco SPME fiber (50/30 *μ*m DVB/CAR/PDMS). After the equilibration period, the fiber was exposed to the sample headspace for 25 min at 40°C. Desorption of the analytes was then carried out in the GC injector at 250°C under splitless conditions. The apparatus was a PerkinElmer Clarus 500 GC–MS system (Waltham, Massachusetts, United States) equipped with a flame ionization detector (FID). A Varian Factor Four VF‐5 was used to separate the analytes. The separation of the analyses was achieved following the temperature program: 45°C held for 2.0 min and then increased by 6°C per minute up to 220° for 10 min. The mass spectrometer was operated at 70 eV (EI) in full scan mode in the range 35–450 m/z. The ion source and the connection parts temperature were set at 180°C and 200°C, respectively. The identification of the analytes was obtained by comparing the mass spectra with those reported in the spectral databases. Further linear retention indices (LRIs) were calculated and compared with values from the literature. Quantification was performed based on FID peak areas and the relative amounts were expressed as percentages. Each analysis was conducted in triplicate to ensure reproducibility.

### 2.8. Statistical Analysis

The statistical analysis was performed with the XLSTAT 2025.1.1 software (Addinsoft SARL, New York, United States) using a one‐way analysis of variance. To describe statistical differences between means at a significance level of *p* < 0.05, Fisher′s least significant difference test was used.

## 3. Results and Discussion

### 3.1. Morphological Properties of the Sweet Cherry Samples

The examination of the physical properties of the samples produced a series of intriguing findings. The results of the analysis of morphological traits are given in Table 1.

**Table 1:** Morphological properties of the sweet cherry samples.

Samples	Weight (g)	Length (mm)	Width (mm)	Thickness (mm)	Geometric mean diameter (mm)	Surface area (mm^2^)	Sphericity (%)	Volume (mm^3^)
R	9.025 ± 1.55^a^	2.200 ± 0.21^a^	2.525 ± 0.41^a^	1.896 ± 0.09^a^	2.190 ± 0.23^a^	15.065 ± 2.90^a^	0.996 ± 0.02^a^	5.511 ± 1.43^a^
G	6.025 ± 1.52^b^	1.813 ± 0.20^b^	1.695 ± 0.43^b^	1.715 ± 0.07^b^	1.739 ± 0.25^b^	9.496 ± 2.88^b^	0.960 ± 0.02^b^	2.756 ± 1.40^b^
Pr > *F* (Model)	< 0.0001	< 0.0001	< 0.0001	< 0.0001	< 0.0001	< 0.0001	0.004	< 0.0001
Significant	Yes	Yes	Yes	Yes	Yes	Yes	Yes	Yes

*Note:* Means that show different letters are significantly different (*p* < 0.05).

Abbreviations: G, “Graffione” sweet cherry *cv*; R, “Ravenna a gambo lungo” sweet cherry *cv*.

There was a significant difference in average fruit weight between the two cultivars, with a value of 9.025 ± 1.55 g for the “Ravenna a gambo lungo” sweet cherry cultivar, whereas the weight for the “Graffione” sweet cherry cultivar was lower at 6.025 ± 1.52 g. Cherries of the “Graffione” cv. are medium‐sized, whereas the fruit of “Ravenna a gambo lungo” is considered to be large‐sized [22]. Consumers are known to be drawn to sweet cherries that are large in both size and weight of fruit. The “Ravenna a gambo lungo” sweet cherry *cv* had length, width and thickness values (2.200, 2.525, and 1.896 mm, respectively) that were higher than those of the “Graffione” sweet cherry *cv* sample (1.813, 1.695, and 1.715 mm, respectively). On the other hand, a characterization of autochthonous Campania cherries carried out by Di Matteo et al. [10] concluded that the morphometric parameters are dependent on the soil and pedoclimatic conditions. Researchers have stated that the size of sweet cherries is the main factor in determining their commercial value [23, 24]. Consumers are mostly drawn to large, dark cherries. In terms of sphericity, both studied cultivars have rounded shapes, with values of 0.996 ± 0.02 and 0.960 ± 0.02 for “Ravenna a gambo lungo” and “Graffione,” respectively.

### 3.2. Sensory Analysis

Information about the sensory traits of sweet cherries of the “Graffione” and “Ravenna a gambo lungo” cv. can be found in Table 2.

**Table 2:** Least squares means of sensory descriptors and overall judgments for the sweet cherry sample.

Sample	Color intensity	Crunchiness	Succulence	Consistency of pulp	Sweetness	Bitterness	Astringent	Cherry aroma	Plum aroma	Apple aroma	Floral aroma	Aromatic intensity	Overall judgment
R	8.96 ± 0.12^a^	8.00 ± 1.06^a^	8.92 ± 0.51^a^	8.02 ± 0.33^a^	7.42 ± 0.54^b^	0.35 ± 0.19^a^	1.32 ± 0.67^a^	9.08 ± 0.53^a^	8.03 ± 0.52^b^	1.95 ± 0.50^a^	3.43 ± 1.01^a^	9.09 ± 0.34^a^	9.07 ± 0.52^a^
G	8.92 ± 0.11^a^	6.72 ± 1.05^b^	7.92 ± 0.50^b^	7.39 ± 0.30^b^	8.48 ± 0.55^a^	0.00 ± 0.00_b_	0.00 ± 0.00^b^	8.05 ± 0.50^b^	9.05 ± 0.53^a^	0.97 ± 0.52^b^	1.43 ± 1.05^b^	8.45 ± 0.35^b^	8.05 ± 0.50^b^
Pr > *F* (Model)	0.240	< 0.0001	< 0.0001	< 0.0001	< 0.0001	< 0.0001	< 0.0001	< 0.0001	< 0.0001	< 0.0001	< 0.0001	< 0.0001	< 0.0001
Significant	No	Yes	Yes	Yes	Yes	Yes	Yes	Yes	Yes	Yes	Yes	Yes	Yes

*Note:* Means that show different letters are significantly different (*p* < 0.05).

Abbreviations: G, “Graffione” sweet cherry cv; R, “Ravenna a gambo lungo” sweet cherry cv.

**Figure 2:**
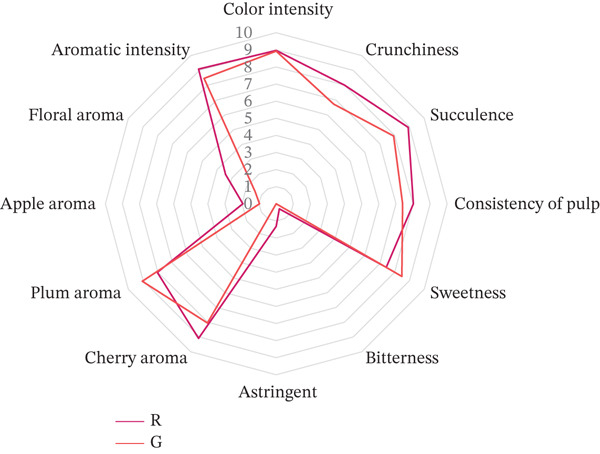
Radar chart of the analyzed sweet cherry samples. G, “Graffione” sweet cherry cv; R, “Ravenna a gambo lungo” sweet cherry *cv*.

The most notable aspect is the complexity of their aromatic properties. In particular, the “Ravenna a gambo lungo” cv. had an intensity of crunchiness of 8.00 ± 1.06, which was higher than the “Graffione” cv. at 6.72 ± 1.05. The crunchiness of the fruit is especially significant as it has a considerable impact on its quality. This is because it produces a distinctive noise, which is seen as a positive sensation and is associated with quality, freshness, and listening pleasure [25, 26]. When compared with the “Graffione” sweet cherry, the “Ravenna a gambo lungo” cherry exhibited high levels of succulence (8.92 ± 0.51), consistency of pulp (8.02 ± 0.33), cherry aroma (9.08 ± 0.53), apple aroma (1.95 ± 0.50), floral aroma (3.43 ± 1.01), and aromatic intensity (9.09 ± 0.34). Conversely, the sweetness (8.48 ± 0.55) and plum aroma (9.05 ± 0.53) of “Graffione” cherries were greater than those of “Ravenna a gambo lungo” cherries (Table 2). Figure 2 shows a radar graph illustrating the sensory profiles of the two sweet cherry cultivars analyzed.

**Figure 3:**
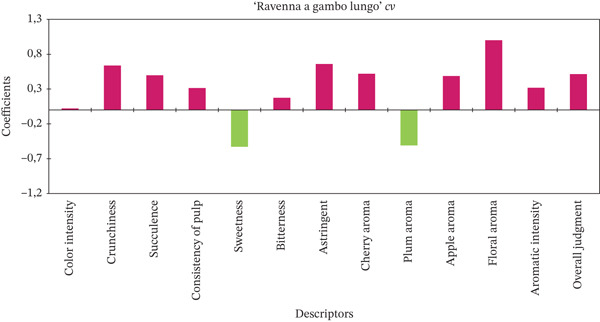
Coefficients of the analysis of variance models of the sweet cherry samples from the “Ravenna a gambo lungo” sweet cherry cv.

Figure 3 shows the coefficients of the analysis of variance models of the sweet cherry samples from the “Ravenna a gambo lungo” sweet cherry cultivar. The magenta color is linked to coefficients with a significant positive value, whereas the green color is connected to coefficients with a significant negative value. It is evident that the cherries under scrutiny are distinguished by their crunchiness and the aromas of floral, cherry, and apple.

**Figure 4:**
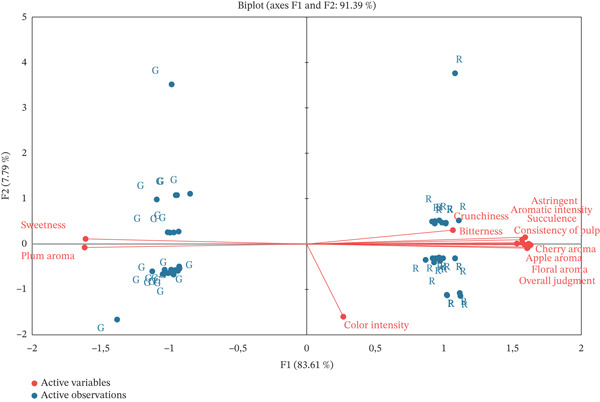
PCA loading diagram illustrating the multivariate dispersion among the sweet cherry samples. G, “Graffione” sweet cherry cv; R, “Ravenna a gambo lungo” sweet cherry *cv*.

To further differentiate the two cherry cultivars, principal component analysis (PCA) was applied to evaluate the multivariate distribution of the samples. As illustrated in Figure 4, clear sensory distinctions between the cultivars can be observed. The PCA model was constructed using the median values of each sensory parameter. The first two principal components accounted for 91.39% of the total variance, with F1 explaining 83.61% and F2 contributing 7.79%. The score plot shows that the samples are separated into distinct quadrants. In particular, the “Ravenna a gambo lungo” (R) samples are located on the right‐hand side of the projection (Quadrants I and II) and are associated with attributes such as crunchiness, cherry, and floral aromas, apple notes, sweetness, and pulp consistency.

The cherries of the cultivar “Graffione” (G) are to be found in the third and fourth quadrants to the left of the PCA projection and are distinguished by their sweetness and plum aroma. The graphs reveal that cherries can be distinguished using sensory descriptors. Although both cultivars studied received very high scores in the overall judgment, the cultivar “Ravenna a gambo lungo” obtained a higher score (9.07 ± 0.52) than the “Graffione” (8.05 ± 0.50). The performance of the sensory analysis panel is crucial for obtaining reliable results, since it is the panelists who determine the outcome of the assessment. For this reason, the performance of the assessors conducting the sensory analysis in this research was investigated. Figure 3 presents the mean ranking of the effects attributed to each assessor across all evaluated attributes. A “+” symbol indicates that a panelist was able to discriminate between samples and showed agreement with the panel consensus, whereas a “−” denotes a lack of agreement. Overall, the assessors demonstrated a high level of performance, with nearly all panelists successfully differentiating between the two cultivars and showing strong consistency with the group (as indicated by the green‐highlighted cells).

**Figure 5:**
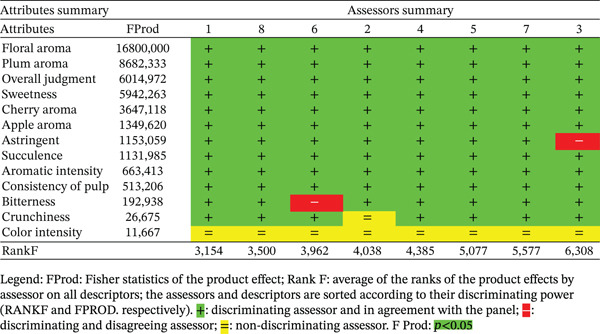
Control of assessor performance in the sensorial analysis. Legend: FProd: Fisher statistics of the product effect; Rank F: average of the ranks of the product effects by assessor on all descriptors; the assessors and descriptors are sorted according to their discriminating power (RANKF and FPROD., respectively). +: discriminating assessor and in agreement with the panel; −: discriminating and disagreeing assessor; =: nondiscriminating assessor. FProd: *p* < 0.05.

The panelist in cell n.3 (red cell) did not agree with the rest of the panel when it came to the astringent descriptor. On the other hand, panelist n.6 is indicated in a red box because, despite being discriminatory for the bitterness descriptor, he disagreed with the panel. The sequence of the panelists in Figure 5 (based on RankF) demonstrates that panelist n.1 exhibited the greatest discriminatory capacity with respect to all attributes, whereas panelist n.3 demonstrated the least discriminatory capacity. Regarding the FProd values, it can be seen that the panel discriminated against all attributes (green cells). Overall, our findings demonstrate that the experts agreed with the rest of the panel to a high degree. Moreover, Peltier et al. [27] emphasized that the reliability of the entire panel remains unaffected even when some judges disagree.

### 3.3. TAC

The findings relating to the levels of TPC and antioxidant capacity in “Ravenna a gambo lungo” and “Graffione” sweet cherries are presented in Table 3.

**Table 3:** Total phenolic content (TPC) expressed as mg of gallic acid equivalents (GAE/g); ferric reducing antioxidant power assay (FRAP); ABTS^•+^ radical scavenging activity on edible part.

Samples	TPC (mg GAE/g)	FRAP (mmol Fe^2+^/g)	ABTS^•+^ (mmol TE/g)
Graffione	3.26 ± 0.78	2.14 ± 0.38	1.25 ± 0.02
Ravenna a gambo lungo	3.27 ± 1.19	2.26 ± 0.78	1.20 ± 0.25
*p* value	0.994	0.820	0.799

*Note:* Data represents mean ± standard deviation of *n* = 3 biological replicates and *n* = 2 technical replicates.

As numerous studies [4, 28, 29] have reported, there is a close relationship between antioxidant capacity and phenolic content. These are directly linked to the health benefits of cherries [30]. Interestingly, the antioxidant heritage in the samples analyzed is higher than that found in sweet cherries by other authors [29]. There were no significant differences between the two samples. The values for the “Ravenna a gambo lungo” sample were TPC (3.27 mg GAE/g), ABTS^•+^ (1.20 mmol TE/g), and FRAP (2.26 mmol Fe^2+^/g). For the “Graffione” sample, they were TPC (3.26 mg GAE/g), ABTS^•+^ (1.25 mmol TE/g), and FRAP (2.14 mmol Fe^2+^/g) (Table 4). These results highlight the high levels of TPC found in local sweet cherries from the Celleno area. These levels are higher than those reported by Clodoveo et al. [29] for other varieties. The authors discovered 1.22 mg GAE/g in Ferrovia cv., 2.4 mg GAE/g in Sweetheart *cv.,* and 2.1 mg GAE/g in Lapins cv. Another study by Hu et al. [31] on Australian sweet cherry varieties also found that TPC (from 0.87 to 1.73 mg gallic acid equivalents/g), antioxidant capacity (ABTS^•+^ from 0.37 to 0.51 mg ascorbic acid equivalents/g), and FRAP (from 0.63 to 0.95 mg ascorbic acid equivalents/g) were lower than in our study.

**Table 4:** Volatile content (percentage mean value ± standard deviation) of cherries, as determined by HS‐SPME/GC–MS.

N°	COMPONENT^d^	LRI^e^	LRI^f^	G (%)	*R*(%)
1	Hexanal	822	819	16.4 ± 1.4^a^	3.8 ± 0.06^b^
2	*Trans*‐2‐hexenal	827	824	9.0 ± 0.13^a^	2.5 ± 0.07^b^
3	*Trans*‐2‐hexenol	871	879	2.5 ± 0.07^a^	38.9 ± 3.10^b^
4	*β*‐Thujene	962	968	—	1.0 ± 0.03
5	Benzaldheyde	991	996	1.5 ± 0.04^a^	7.5 ± 0.10^b^
6	Limonene	1032	1029	2.3 ± 0.06^a^	2.4 ± 0.07^a^
7	1,8‐Cineole	1035	1033	6.9 ± 0.09	tr
8	Linalool	1096	1092	24.7 ± 1.55^a^	1.3 ± 0.05^b^
9	Nonanal	1100	1104	3.1 ± 0.06^a^	8.9 ± 0.12^b^
10	Terpinen‐4‐ol	1178	1182	3.4 ± 0.07^a^	1.9 ± 0.07^b^
11	Decanal	1181	1184	11.7 ± 1.05^a^	22.1 ± 2.10^b^
12	*α-*Terpineol	1189	1193	3.6 ± 0.07	—
13	Linalyl acetate	1247	1252	12.4 ± 0.62	—
14	L‐Bornyl acetate	1281	1284	2.0 ± 0.04	—
15	*Cis*‐geranylacetone	1430	1427	Tr	6.3 ± 0.10
16	Nerolidol	1571	1565	—	1.9 ± 0.05
	SUM			99.5	98.5

*Note:* Data are means ± standard deviation of three (*n* = 3) replicates. Means with different letters among the columns, are significant different (ANOVA test followed by Tukey′s HSD test, *p* < 0.01). Tr: percentage mean values < 0.1*%*; —: not detected.

Abbreviations: G, “Graffione” sweet cherry *cv;* R, “Ravenna a Gambo lungo” sweet cherry *cv*.

^d^The components are reported according to their elution order on apolar column.

^e^Linear retention indices measured on apolar column.

^f^Linear retention indices from literature.

### 3.4. Volatile Fraction

The performed analyses revealed that the volatile fraction of the analyzed cherries was characterized by the presence of volatile compounds, which were distributed differently between the two samples (Table 4).

Specifically, in “Ravenna a gambo lungo” cherries, the main volatiles were *trans*‐2‐hexenol (38.9%), decanal (22.1%), nonanal (8.9%), benzaldehyde (7.5%), and *cis*‐geranyl acetone (6.3%). According to Villavicencio et al. [32], the main descriptors of these compounds are trans‐2‐hexenol: fruity and green; decanal: citrus and floral; nonanal: apple, fruity, citrus, and floral; benzaldehyde: almond and cherry; cis‐geranyl acetone: floral and rose. Of the volatile compounds detected in “Graffione,” linalool (24.7%) and hexanal (16.4%) were the most abundant. However, what sets this cultivar apart is the presence of *trans*‐2‐hexenal (9.0%) and 1,8‐cineole (6.9%). The main descriptors of these compounds have been identified [33] as hexanal: green and fruity; *trans*‐2‐hexenal: plum and vegetable; linalool: fruity and grape; 1,8‐cineole: herbaceous and spicy. These findings are in agreement with those obtained by sensory analysis (Table 2). The sensory panel of trained judges confirmed the different profiles of the two sweet cherry cultivars studied. The “Ravenna a gambo lungo” sample showed higher values for the descriptors of cherry (9.08), apple (1.95), and floral (3.43) aromas in comparison with “Graffione” cv. These are, in fact, linked to the volatile compounds most prevalent in the “Ravenna a gambo lungo” sample: *trans*‐2‐hexenol (fruity and green), decanal (citrus and floral), nonanal (apple, fruity, citrus, and floral), and benzaldehyde (almond and cherry). Another volatile compound present in the “Ravenna a gambo lungo” sample is *cis*‐geranyl acetone, which has a floral and rose aroma.

Regarding the sample “Graffione,” the sensory analysis (Table 2) revealed a higher plum aroma (9.05), which is associated with the volatile compound *trans*‐2‐hexenal, responsible for the plum and vegetal notes. Finally, other qualitative differences were observable; *α*‐terpineol, linalyl acetate, and L‐bornyl acetate were found in “Graffione” but were absent in “Ravenna a gambo lungo,” whereas *β*‐thujene and nerolidol were present only in “Ravenna a gambo lungo” samples. The literature [32–36] emphasized that the presence of volatile compounds is closely linked to the cultivar, determining the final perceived aroma.

## 4. Conclusions

This is the first study to look at the nutritional and sensory properties of two sweet cherry cultivars grown in the Celleno area of central Italy. It gives information about the local and more popular types of sweet cherry that consumers like. In conclusion, this study found that the local cherry cultivars “Ravenna a gambo lungo” and “Graffione” had a high TPC and great antioxidant capacity. Their sensory profiles were characterized by different descriptors for each cultivar. These results are consistent with the differences observed in the volatile profiles of “Ravenna a gambo lungo” and “Graffione.” Our data will provide greater insight into the excellent quality of the sweet cherries, which play an important role in Lazio′s traditional heritage. It is therefore worth noting that the native cherries we analyzed, thanks to their high antioxidant capacity, constitute a valuable asset in the panorama of quality production, also supporting environmental sustainability. Nevertheless, we are aware of the limitations of the antioxidant methods used. For example, interference from other reactive compounds may result in an overestimation of antioxidant activity. Further research is therefore needed to address these limitations and improve the accuracy of the evaluation methods.

## Funding

Open access publishing was facilitated by the Universita degli Studi della Tuscia, as part of the Wiley‐CRUI‐CARE agreement.

## Ethics Statement

The authors have nothing to report.

## Conflicts of Interest

The authors declare no conflicts of interest.

## Data Availability

The data that support the findings of this study are available from the corresponding author upon reasonable request.
